# Exploring causal mechanisms and essential factors in the prevention and control of chlamydia in Guangdong, Southern China: a study protocol

**DOI:** 10.1186/s12879-023-08597-y

**Published:** 2023-09-25

**Authors:** Wenjun He, Peizhen Zhao, Tao Yang, Yuting Wan, Dadong Wu, Xiaoshan Chen, Zizhen Huang, Huanyuan Luo, Dong (Roman) Xu, Shujie Huang, Cheng Wang

**Affiliations:** 1 School of Public Health, Southern Medical University, Guangzhou, China; 2 Acacia Lab for Implementation Science, School of Health Management, Southern Medical University, Guangzhou, China; 3 Dermatology Hospital, Southern Medical University, Guangzhou, China; 4 Southern Medical University Institute for Global Health, Guangzhou, China; 5 School of Public Health, the Key Laboratory of Environmental Pollution Monitoring and Disease Control, Ministry of Education, Guizhou Medical University, Guiyang, China; 6 School of Health Management, Southern Medical University, Guangzhou, China; 7 Affiliated Shenzhen Maternity & Child Healthcare Hospital, Southern Medical University, Shenzhen, China; 8 Acacia Lab for Implementation Science, School of Health Management and Dermatology Hospital, Southern Medical University, Guangzhou, China; 9 Center for World Health Organization Studies and Department of Health Management, School of Health Management, Southern Medical University, Guangzhou, China; 10 School of Health Management and Dermatology Hospital, Southern Medical University, Guangzhou, China; 11 Center for World Health Organization Studies, Southern Medical University, Guangzhou, China; 12 Acacia Lab for Implementation Science, Southern Medical University Institute for Global Health (SIGHT), Guangzhou, China; 13 Acacia Lab for Implementation Science, School of Public Health, Southern Medical University, Guangzhou, China

**Keywords:** Chlamydia prevention and control, Causal loop diagram, System dynamics modeling, Intervention development, Feedback mechanisms

## Abstract

**Background:**

Chlamydia Trachomatis (CT) is among the most prevalent sexually transmitted diseases (STDs) globally. According to the World Health Organization, more than 131 million people get infected with CT annually. CT is usually transmitted via sexual contact or perinatal exposure and can result in severe long-term complications. In developing nations, particularly, the prevention and control of CT is challenging. Hence, this study will explore the feedback mechanisms of chlamydia prevention and control, as well as identify the essential factors affecting the control and prevention of this infection in China.

**Methods:**

Our study will employ a mixed-methods research design that encompasses both qualitative and quantitative methods. Firstly, we will develop a causal loop diagram (CLD) based on the literature review and optimize it via in-depth interviews with stakeholders. Additionally, we will utilize a quantitative method called MICMAC(Impact Matrix Cross-Reference Multiplication Applied to a Classification tool) to obtain consensus among different stakeholders and pinpoint the key information. Next, the CLD will be transformed into a system dynamics model (SDM) to evaluate the feedback mechanisms within the CLD. The causality in the CLD will be modeled using mathematical equations, which facilitate the transformation into an SDM. As such, we will be able to analyze the dynamic behavior of the system and its response to different decisions.

**Discussion:**

Our study offers a systematic perspective on the control and prevention of chlamydia infection through system dynamics modeling, examining the dynamic properties and background factors of the system. The creation of the CLD affords stakeholders the chance to comprehend the functionality of their relationships and improve cooperation. Consequently, by evaluating the outcomes of these simulations, it will be possible to analyze and determine potential interventions and their effects on chlamydia infections. This modeling approach can help us gain insight into the dynamic characteristics of the system, evaluate the potential outcomes of different decisions, and design control strategies to either stabilize the system or adjust its behavior.

## Background

### Global burden of disease due to chlamydia infection

Genital Chlamydia Trachomatis (CT) stands out as one of the most widespread sexually transmitted diseases (STDs) globally. The World Health Organization approximated that CT infection affects over 131 million individuals annually [1]. CT infection mostly spreads via sexual contact or perinatal routes. The majority of carriers display subtle symptoms, and recurrent or persistent infections could give rise to pelvic inflammatory disease (PID) and long-term complications in women [2] as well as urethritis in men. It also increases the risk of uterine cancer [3, 4]. Additionally, infection with the human immunodeficiency virus (HIV) is 2–3 times more likely [5]. CT infection not only jeopardizes the sexual and reproductive wellbeing of patients but also imposes a significant public health burden [6, 7].

In the global population (aged between 15 and 49 years), the rate of CT infection in women is 3.8% compared to 2.7% in men. Women are more susceptible to CT infection [8]. The prevalence of genital CT infection varies broadly across countries and regions, and previous research has suggested that it could be associated with differences in social environment, cultural background, economic levels, and sexual beliefs in different areas [1, 9].

### Chlamydia infection prevention and treatment: the challenges in China

Antibiotics, such as azithromycin, doxycycline, and metronidazole, are typically effective in treating CT infection, especially when administered promptly [10]. A study analyzing case data from 105 sexually transmitted disease (STD) sentinel sites in China revealed a growing reported incidence of CT infection that peaked at a rate of 55.32/100,000 in 2019, with an annual increase of 10.44% [11]. It is worth noting that estimating the burden of genital CT infection in China is mainly based on the STD surveillance system, which could be affected by various factors.

Preventing and controlling infectious diseases entails addressing three critical aspects: managing the source, interrupting the transmission route, and protecting susceptible populations. In the context of sexual transmission, which remains a primary concern, there are no effective vaccines for CT infection. Nearly 80% of genital CT carriers are asymptomatic [12], underscoring the importance of early screening and treatment as vital interventional measures to manage CT infection and forestall serious complications.

To effectively contain the epidemic of chlamydial infection in the reproductive tract and the resulting adverse health outcomes, the Guangdong Provincial Health and Wellness Commission has launched the “Pilot Project for Prevention and Control of Chlamydial Infection in Guangdong Province (2022–2025) Program.“

### Previous research on the CT prevention and treatment: key limitations

Screening for genital CT infection and early treatment plays a crucial role in controlling the spread of CT infection and preventing serious complications. In many countries, screening for genital CT infection is often recommended for young people aged 25 or younger, with screening frequencies ranging from once a year to once every two years [13–17]. The implementation of these screening programs has played a significant role in reducing the spread of genital CT infection in many countries. It is worth noting that previous research had identified people with chlamydia primarily through screening, but their implementation of screening has varied widely.

To achieve a more comprehensive understanding of the screening and treatment process in the broader context, the Guangdong Province in China has launched a pilot project employing the “Three Combos, Three Systems, Three Types of Population, and One Standard” strategy. Using system dynamics, stakeholders can comprehend their role and function in the overall system and the linkages with other stakeholders. Additionally, researchers can identify key factors that influence Chlamydia infection management and develop systemic interventions. System dynamics is an indispensable tool for studying complex problems by combining feedback control principles with causal analysis methods. Relationships between different subsystems or parts of a system are often nonlinear and characterized by delay. A disturbance in one part of the system could result in changes in other parts. The causal feedback loops in the system provide a powerful method for understanding and solving complex health issues.

In the health field, system dynamics built using a mixed-methods approach can better reflect the complexity and mechanisms of the chlamydia prevention and control system. Dhirasasna and Sahin combined quantitative and qualitative methods to choose stakeholders, identify endogenous and exogenous variables, and develop causal loop diagrams (CLDs) [18]. The traditional approach to constructing CLDs involves qualitative methods like literature review, observation, and stakeholder’ interviews. Mixed method research is crucial [19] in comprehending the problem from multiple angles and gaining a more comprehensive understanding of the issue while quantifying structures that are difficult to measure.

Thompson and Tebbens demonstrated in 2008 how system dynamics could significantly impact global health policy analysis by reducing the global caseload of smallpox to just 1000 cases every year [20]. This example showcases the impact system dynamics can have on health policy analysis worldwide. System dynamics, which was introduced in China in the 1980s, has been deeply applied in areas like regional economic development and urban planning [18, 21, 22]. Influential domestic and international organizations have encouraged and supported the application of system dynamics to understand the causes and trends of diseases, prevention design, treatments, and policy interventions [19].

## Study aim

This study aims to collate the viewpoints of various stakeholders with the objective of unraveling the intricate feedback mechanisms involved in chlamydia prevention and control. Concurrently, it seeks to identify the pivotal factors that significantly influence the prevention and mitigation of chlamydia infection.

## Study design

Our study will utilize a constructed causal loop diagram (CLD), optimized by stakeholders, and a hybrid study design that incorporates both quantitative and qualitative methods with dynamic simulation modeling to investigate supportive systems for chlamydia trachomatis prevention and control. Initially, a literature review will be conducted to outline the causal feedback mechanism of chlamydia trachomatis prevention and control in China. Subsequently, stakeholders from various regions of Guangdong Province, including the Center for Disease Control and Prevention, healthcare institutions, the population, and the government, will participate in in-depth interviews to optimize specific variables in the feedback mechanism of chlamydia trachomatis infection and improve the scientific nature of the mechanism. Finally, dynamic simulation modeling will be used to evaluate the effectiveness of potential chlamydia trachomatis prevention and control strategies. A representative sample of five cities in Guangdong Province, Shenzhen, Zhuhai, Maoming, Jieyang, and Yunfu, and stakeholders from these localities, will be selected.

### Construction of causal loop diagram

At this stage, a CLD will be developed through a literature review. It will provide a visual representation of a system, including its boundaries, components, and the relationships within it. For this study, the bounded system is Chlamydia trachomatis infection and its impact on healthcare institutions, practices, and interventions. A systematic review by Negar Darabia [23] presented an example of the basic structure of a population-level infectious disease model, with the loop “R” representing an enhanced loop and loop “B” representing a balanced loop. The CLD consists of variables (system factors) and links (relationships). Links are represented by arrows and the type of relationship is denoted by a positive (+) or negative (-) sign. System variables and links are matched to form feedback loops, which reflect their relationships. Feedback loops can reinforce or balance. An enhanced loop represents a change that produces more of the same, leading to an increase or decrease in the magnitude of the response. A balanced loop demonstrates how a change in one direction is balanced by a change in the opposite direction.

### Optimization of causal loop diagram

A preliminary version of the CLD will be developed through a literature review. Then it will be optimized through a hybrid approach involving both quantitative and qualitative methods. Given the challenges of organizing face-to-face meetings with stakeholders, online meetings will be used to optimize the CLD, particularly the stakeholders coming from diverse cities and various professions. The preliminary version of the CLD will be divided into four subsystems, namely CDC, healthcare institutions, the population, and government, and different stakeholders will be invited to modify their corresponding subsystems through online deep interviews. A non-structured, direct, one-on-one interview format will be used to gather information on stakeholders’ potential motives, attitudes, and emotions related to a particular issue or behavior. This information will be used to develop strategies for addressing the specific issue.

Structural analysis-MICMAC [24], an Impact Matrix Cross-Reference Multiplication Applied to a Classification tool, will be used to quantitatively evaluate the strength, direct or indirect relationship, and dependence among variables and stakeholders. Experts will rate the relationships between variables on a scale of zero (no influence), one (weak influence), two (moderate influence), and three (strong influence). Variables will be selected from the list of stakeholders’ variables, and important relationships will be quantified by removing weak connections and achieving consensus among different stakeholders. The information obtained through the CLD and MICMAC will be essential and valuable for decision-makers.

### Research team roles

The development of a CLD entails collaboration from a team, and outlining the roles and responsibilities of each member is paramount. Each team member has distinct roles and duties as outlined below (although in some cases, one person may assume multiple roles):

#### Interviewers

Consisting of a lead interviewer and a supporting interviewer, the former follows a standard interview outline prepared beforehand, inquiring with questions in a prescribed order, responding to interviewees’ responses, and modifying the interview’s course as necessary. The supporting interviewer offers stimulation and concerns based on the interviewees’ responses.

#### Modelers

Modelers listen to the interviews and adapt the system dynamics model accordingly. They do not partake in discussions. In this study, all the research team members are modelers.

#### Organizers

These individuals schedule online meetings and extend invitations to relevant stakeholders to participate. They also ensure the online meetings run without a hitch by resolving technical difficulties such as issues with video conferencing software.

#### Technicians

Their responsibility is to ensure that online meetings run smoothly. They assist attendees with technical challenges during the meetings, such as setting up equipment, creating meeting links, and supervising activities during the meetings. They also facilitate group votes and keep meeting minutes.

#### Research technology consultants

Their duties include updating the interview outline, assisting with fieldwork, and providing professional recommendations on interview techniques.

## Action plan for constructing CLD

**Fig. 1:**
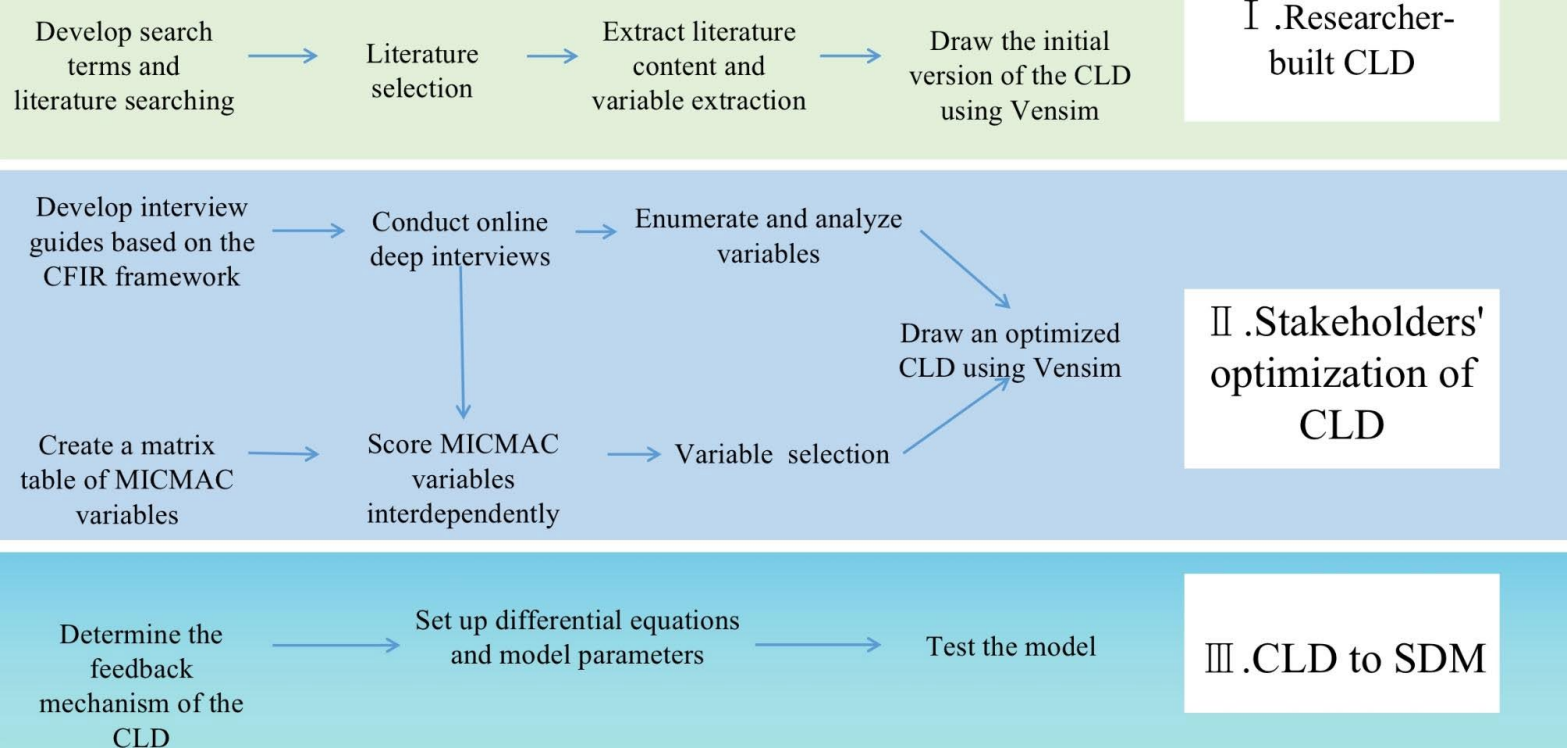
Action Plan for Constructing CLD

The process of constructing a CLD is portrayed in Fig. 1, covering three primary stages: researcher-built CLD, optimization of CLD by stakeholders, and the conversion of CLD to SDM. Each stage represents a series of sequential steps. Throughout the modeling and optimization phase, both the researchers and stakeholders can enhance their perception of complex problems.

### Researcher-built CLD

#### Literature searching

At this stage, articles on chlamydia infection and control that employ methods related to CLD will be retrieved. This strategy will be executed by exploring six databases, including China Knowledge Network, Wanfang Data, China Scientific Journal Database, Web of Science, SinoMed, and PubMed, for both Chinese and foreign-language articles using a combination of search terms(**#1** AND (**#2** OR **#3**)). The search terms are as follows (will be optimized in practice):

**#1**“Systems Analysis” “System Dynamic*” “Agent-Based Model*” “Agent Based Model*” “causal loop diagrams”.

**#2**“Chlamydia/analysis” “Chlamydia/epidemiology” “Chlamydia/statistics numerical data” “Chlamydia Infections/analysis” “Chlamydia Infections/complications” “Chlamydia Infections/diagnosis” “Chlamydia Infections/economics” “Chlamydia Infections/epidemiology” “Chlamydia Infections/statistics and numerical data” “Infection, Chlamydia” “Chlamydia Infection”.

**#3**“sexually transmitted diseases/analysis” “sexually transmitted diseases/complications” “sexually transmitted diseases/economics” “sexually transmitted diseases/epidemiology” “sexually transmitted diseases/prevention and control” “sexually transmitted diseases/statistics and numerical data” “Sexually Transmitted disease*” “Venereal Disease” “STD” “STI*” “Sexually Transmitted Infection*”.

#### Literature selection

After literature searching, all selected literature will be uploaded to EndNote software, and the duplicates will be removed. Then inclusion and exclusion criteria will be applied to each article. Titles, keywords, and abstracts will be perused to comprehend the research content, findings, and overall conclusions. Any disagreements during the review process will be settled through consultation and discussion among reviewers.

Inclusion criteria: Studies on sexually transmitted infections, specifically the transmission, treatment, prevention, and policies related to CT infection. Preferably, these studies were presented in the form of causal loop diagrams, system dynamics, or systems thinking.

Exclusion criteria: Duplicate; Articles inaccessible in full text; Non-peer-reviewed articles (such as conference papers and preprints); Articles focused exclusively on the pathological mechanisms of CT infection; Studies on other diseases such as blinding trachoma.

This study will not conduct any quality assessments; hence, the inclusion criteria is broad, and the articles will be chosen varied in quality.

#### Variable extraction

To create the initial version of the CLD, the researchers divided it into a Susceptible-Infected-Recovered (SIR) model and four subsystems: disease control, healthcare institutions, populations, and policy/governments. A data extraction table will be generated based on the four subsystems, and the literature will be organized into the proper system while performing variable extraction at the same time. The acquired information will be kept in a database for future reference and analysis.

Using the SIR model main framework, the variables will be included in an online shared PowerPoint, and multiple researchers will participate in drawing the CLD sketch collectively through the shared PowerPoint. After that, researchers will utilize the Vensim software to draw the standard CLD.

### Stakeholders’ optimization of CLD

#### Qualitative study

To better comprehend the perspectives of stakeholders on CT prevention and treatment, qualitative interviews will be utilized to collect their opinions. A semi-structured approach will be taken to develop the interview framework. The Consolidated Framework for Implementation Research (CFIR) will be utilized to construct a structured interview framework based on the obstacles and facilitators of CT prevention and treatment. Moreover, open-ended questions will be included to ensure all stakeholders’ thoughts are taken into consideration. Identifying stakeholders(as interviewees) is a crucial step in this study. The four subsystems of the initial version of the CLD: disease control, healthcare institutions, populations, and governments, will be utilized to identify stakeholders. At least one or two stakeholders will be identified from each subsystem. Experts in the field, keynote speakers, and authors of articles with significant influence, will be used to identify stakeholders. Those who exert the greatest impact or are most easily influenced by CT prevention and treatment policies will be selected. Interviewers will be selected based on their experience and knowledge in the field. Before the interview, interviewers will be trained, and after training, they will participate in an online simulation to assess their skills. Prior to the online interview, the interviewees will be asked to sign an informed consent form. To ensure quality control, the lead interviewers will collaborate with a supporting interviewer. The CFIR coding manual will be used by interviewers to code the interview transcripts and divide the codes into general categories and subcategories. Through inductive and deductive analysis, the barriers and facilitators of CT infection screening and treatment protocols will be identified. Healthcare institution administrative personnel, healthcare personnel, and patients will be interviewed for this study (refer to Table 1).

**Table 1:** Criteria for inclusion and exclusion of interviewees

Interviewee types	Inclusion criteria	Exclusion criteria
Administrative personnel	• Served in management positions in healthcare departments (such as health commissioner, hospital dean, maternity hospital dean, public health center director, and disease control and prevention center director); • Responsible for overseeing or implementing measures to prevent and control Chlamydia infection; • With at least 2 years of experience in their current position.	• Appointed to manage positions in healthcare departments; • Managers who cannot complete the survey interviews for various reasons, including work.
Healthcare professionals	• Have a medical background and work in relevant departments (such as infection control departments, preventive medicine departments, dermatology and ophthalmology departments, maternity and pediatric departments, and nurses); • With at least 2 years of experience in their current position.	• Work in relevant departments and are loaned to other departments; • Medical staff who cannot complete the survey interviews for various reasons, including work.
Patients	•Have been diagnosed with Chlamydia infection within the past year and received treatment; • Aged 25–35.	• Have been diagnosed with other diseases, such as AIDS, hepatitis A, and hepatitis B.

#### Quantitative study

The CLD variables will be extracted, merged, plited, by the researchers, resulting in a new variable list (refer to Table 2), including variable names, definitions, and a variable matrix. Once the interview is completed, the research team will send the variable list and variable matrix to the stakeholders via email for individual completion. The stakeholders will then be asked to rate the impact of the variables on one another using a scale of 0 (no impact), 1 (weak impact), 2 (moderate impact), or 3 (strong impact). If stakeholders wish to add their variables, they can do so at the end of the matrix. The stakeholders will have 14 days to complete the questionnaire, and a reminder will be sent on the seventh day. After obtaining the completed survey, the data will be analyzed using the MICMAC software. MICMAC will quantitatively evaluate important relationships, eliminate weak connections, achieve agreement amongst different stakeholders, and grasp the key information. Finally, variables will be selected based on the analysis results.

**Table 2:** Variable List

Stage	Variable name	Variable definition	Reference	Variable classification in MICMAC	Disposal
*M	-	-	-	Target Variable	Remove
**CLD Final	-	-	-	Core Variable	Merge with other variables

*M = MICMAC Stage

**CLD Final = Final inclusion in CLD Stage

## From causal loop diagram (CLD) to a system dynamics model(SDM)

After constructing the CLD and optimizing it using stakeholder feedback, researchers generated a CLD that demonstrates the feedback mechanism for Chlamydia infection. However, to identify the critical factors involved in the feedback process, solely relying on stakeholder optimization is insufficient. Thus, in the next stage of this study, a system dynamics simulation model will be created to test the feedback mechanism in the CLD. The CLD presents the causal relationship between various variables involved in Chlamydia infection prevention and control, which serves as the basis for constructing the stock and flow diagram. The stock and flow diagram depicts the changes in stock over time and is the foundation of the system dynamics model. The R package (deSolve) will be used to simulate the model, which employs the Euler method to resolve differential equations and simulate the model. The feedback mechanism is defined as a set of differential equations, and the results of the model are calculated and displayed. The model will be analyzed to determine the contribution of each feedback mechanism to the simulated behavior. Various tests will be conducted to validate the model and ensure its stability. A system dynamics model is a “black box” model that assumes causality. After pertinent model data is entered, the model can be rerun and the outcomes can be explained. Therefore, the model must be validated, involving parameter validation, extreme case testing, and verification of variable unit dimensions. Once developed, the model’s process and results will be published in a future paper. The simulation results will help analyze and determine potential interventions, as well as evaluate their impact on Chlamydia infection.

## Discussion

Designing effective methods for preventing and controlling CT requires a comprehensive understanding of the system’s dynamic properties and underlying factors while establishing causality between the infection process, impacts, and results. A CLD is an efficient qualitative system modeling technique for this as it identifies how embedded prevention and control elements interact and influence changes in the system. Furthermore, stakeholder collaboration is possible due to the development of the CLD, facilitating understanding of their relationships and enhancing cooperation. When mathematical equations model causality in the CLD, it transforms into a system dynamics model, capable of analyzing the dynamic behavior of the system and its response to different decisions. This modeling technique determines the dynamic characteristics of the system, evaluates the potential consequences of different decisions, and designs control strategies to stabilize or adjust its behavior. This study employs a mixed-methods research design using both qualitative and quantitative methods to comprehend the support system for CT prevention and control. Firstly, a relevant literature search using keywords creates a first-edition CLD based on the literature information. Secondly, qualitative research methods are utilized to conduct qualitative interviews with stakeholders. Subsequently, the first-edition CLD is optimized using the interview information. Lastly, the MICMAC method is used to identify key factors for preventing and controlling CT infection within the CLD. By constructing a system dynamics model using multi-stakeholder participation, the model construction process provides stakeholders with insight into how the system functions.

This study treats CT prevention and control as a systemic issue. By integrating stakeholder perspectives and employing a mixed-methods approach that combines both qualitative and quantitative analyses, the research aims to elucidate the underlying mechanisms and key factors in CT prevention and control. The identification of system bottlenecks serves to guide the optimization and development of prevention and control policies.

## Strengths and limitations of this study

The uniqueness of this study resides in two aspects. Firstly, the use of system dynamics as a technique for project evaluation, and it will examine the practicality and effectiveness of this approach in curbing CT infection. Secondly, MICMAC, a quantitative method for structural analysis, is employed to achieve consensus among different stakeholders and underline essential data. These innovations provide scientific methods for preventing and controlling Chlamydia infection.

Nevertheless, there are certain limitations to this study. Firstly, the genital Chlamydia infection pilot project is not yet entirely executed, and the elementary staff has insufficient knowledge of CT prevention and control, causing inadequate optimization of the CLD. Secondly, participants from the pilot cities involved in the study may experience potential recall bias, compromising the accuracy of their responses. Thirdly, some variables in the final CLD may be subjective and rely on the perceptions of the pilot cities. It may be challenging to determine the quantity and assign initial and final values to these variables.

## Data Availability

The data that support the findings of this study are available from the corresponding author upon reasonable request.

## Abbreviations

CT: chlamydia trachomatis
STDs: sexually transmitted diseases
CLD: causal loop diagram
MICMAC: Impact Matrix Cross-Reference Multiplication Applied to a Classification tool
SDM: system dynamics model
